# Polyphenols and ADPKD: A Further Aid from Nature?

**DOI:** 10.3390/life16061022

**Published:** 2026-06-18

**Authors:** Caterina Carollo, Alessandra Sorce, Maria Elena Ciuppa, Emanuele Cirafici, Nicola Sinatra, Giulio Geraci, Valentina Paternò, Paola Di Carlo, Rosalia Lo Presti, Giuseppe Mulè, Gregorio Caimi

**Affiliations:** 1 Department of Health Promotion, Mother and Child Care, Internal Medicine and Medical Specialties, University of Palermo, 90100 Palermo, Italyemanuele.cirafici@community.unipa.it (E.C.);; 2 UOSD Nefrologia e Dialisi, Ospedale Paolo Borsellino, 91025 Marsala, Italy; 3 Department of Medicine and Surgery, “Kore” University of Enna, 94100 Enna, Italy; 4 Operative Unit of Diabetology, Umberto I Hospital, Provincial Health Authority (ASP) of Enna, 94100 Enna, Italy; 5 Department of Psychology, Educational Science and Human Movement, University of Palermo, 90127 Palermo, Italy

**Keywords:** ADPKD, autosomal dominant polycystic kidney disease, polyphenols, cardiorenal syndrome, resveratrol

## Abstract

Treating autosomal dominant polycystic kidney disease (ADPKD) has always been a challenge because the disease is too complex for single-target drugs, which are often held back by side effects. This narrative review explores a different strategy: using plant-derived polyphenols to target multiple disease pathways at the same time. Looking at research from 2005 to 2026, we break down how key compounds like resveratrol, curcumin, naringenin, quercetin, and epigallocatechin-3-gallate (EGCG) actually work. Preclinical studies show these molecules can slow down cyst growth by tackling inflammation, rapid cell division, and tissue scarring all at once, while also resetting the skewed energy metabolism of cystic cells. Some mechanisms are strikingly specific, such as naringenin’s direct interaction with polycystin-2 and quercetin’s ability to clear senescent cells. Yet, the real-world hurdle is poor absorption; a recent clinical trial with standard curcumin fell short simply because the compound could not reach the kidneys in high enough concentrations. Moving forward, the field needs to focus on testing these compounds in realistic animal models, designing smart nanoformulations to improve bioavailability, and exploring combinations that could safely complement current therapies like tolvaptan.

## 1. Introduction

The search for adjunctive therapies in autosomal dominant polycystic kidney disease (ADPKD) has long been hampered by the disease’s genetic complexity and the limited number of druggable targets. In this context, polyphenols—a broad class of plant-derived bioactive compounds—have attracted growing attention, not least because of their capacity to act on multiple disease-relevant pathways simultaneously. Work from our group has highlighted their role in cardiorenal disease, particularly through the modulation of sirtuins and nicotinamide adenine dinucleotide (NAD^+^)-dependent metabolic pathways [1,2], and the present review asks whether this same mechanistic framework might extend to ADPKD.

ADPKD is the most common inherited kidney disease and a significant cause of end-stage renal failure: it accounts for the fourth most frequent indication for kidney replacement therapy globally and affects an estimated 12 million people, with a point prevalence of 3–5 per 10,000 [3,4,5]. The disease is genetically heterogeneous, though mutations in Polycystic Kidney Disease gene 1 (PKD1) and Polycystic Kidney Disease gene 2 (PKD2) account for the vast majority of cases. For decades, management was essentially supportive; the 2018 Food and Drug Administration (FDA) approval of tolvaptan represented the first real disease-modifying option, though its use is constrained by hepatotoxicity risk and tolerability issues [6]. Crucially, ADPKD is not simply a cyst disease: inflammation and fibrosis actively drive renal function decline, independent of cyst burden [7].

Polyphenols are widely present in fruits, vegetables, tea, and wine and have shown renoprotective properties in hypertension, diabetic nephropathy, and cardiorenal disease—effects attributable primarily to antifibrotic and anti-inflammatory mechanisms [8,9]. Their ability to modulate Sirtuin-1 (SIRT1) and NAD+ metabolism adds a metabolic dimension that is directly relevant to the energy dysregulation observed in cystic epithelia [10].

## 2. Materials and Methods

This narrative review synthesizes current evidence on the potential of polyphenols to modulate the pathophysiological mechanisms driving ADPKD, with a focus on antioxidative, anti-inflammatory, antifibrotic, and metabolic effects.

We searched PubMed and Google Scholar for publications from January 2005 to March 2026, using the following terms, combined with Boolean operators: “polyphenols”, “autosomal dominant polycystic kidney disease”, “ADPKD”, “polycystic kidney disease”, “oxidative stress”, “inflammation”, “fibrosis”, “mTOR”, “sirtuins”, and “cystogenesis”. Eligible studies were original peer-reviewed articles or reviews—including in vivo animal models, in vitro cell cultures, and human clinical studies—published in English and providing mechanistic or clinical evidence on polyphenol–ADPKD pathway interactions. We excluded editorials, letters, conference abstracts without full text, preprints, and studies on polyphenols in unrelated renal conditions without mechanistic relevance to ADPKD. Additional references were identified by manually screening the bibliographies of included papers and relevant prior reviews.

Compounds were selected on mechanistic grounds: inclusion required evidence of activity on at least one core cystogenic pathway—oxidative stress, inflammatory signalling, fibrosis, mTOR/AMPK activation, or cystic epithelial proliferation.

## 3. APKD: Pathogenesis and Molecular Mechanisms

At its core, ADPKD is a disease of defective polycystin function. Loss-of-function mutations in PKD1 (chromosome 16p13.3) account for 78–80% of cases and typically produce a more severe phenotype with earlier onset of renal failure; PKD2 mutations (chromosome 4q22.1) are responsible for roughly 15% of cases and tend to follow a milder course [11]. A smaller proportion of patients carry mutations in GANAB, DNAJB11, ALG8, ALG9, or IFT140, usually presenting with atypical late-onset disease [12,13], and biallelic variants have been documented in early-onset cases [14,15]. The disease is inherited as an autosomal dominant trait with complete penetrance [16], but its clinical expression extends well beyond the kidneys: hepatic cysts, pancreatic involvement, intracranial aneurysms, and cardiac valve abnormalities all contribute meaningfully to morbidity [16].

***Polycystin Biology*:** PC1 is a large transmembrane receptor expressed at the plasma membrane of renal tubular cells, where it associates with E-cadherin and alpha-, beta-, and gamma-catenin, and is found at focal adhesions, desmosomes, and adherent junctions [17]. Its proteolytic cleavage at the GPS site releases an amino-terminal fragment that remains membrane-tethered to the carboxy-terminal portion—a process that controls PC1 biogenesis and trafficking [18,19]. The carboxy-terminal domain interacts with transcriptional activators and repressors [20] and, critically, physically associates with PC2 at the endoplasmic reticulum to form a complex regulating ion transport and intracellular Ca^2+^ signalling [11,20]. When intact, the PC1-PC2 complex suppresses the cilia-dependent signalling pathways that would otherwise promote cyst growth [21]. PC2, encoded by PKD2, is a non-selective cation channel of the TRP family localized at the endoplasmic reticulum, where it interacts with the store-operated channel TRPC1 [22]. Its C-terminal domain contains a coiled-coil region needed for oligomerization and EF-hand motifs essential for calcium-dependent gating; mutations here abolish channel function [23]. The downstream consequence is dysregulated intracellular Ca^2+^ homeostasis, with impaired channel activity and defective control of the inositol trisphosphate and ryanodine receptors [24,25].

***Cyst Initiation and Expansion*:** The most widely accepted model of cyst initiation is the somatic second-hit hypothesis: a germline mutation in one allele is followed by a somatic mutation that eliminates the remaining functional copy, leading to complete polycystin loss in individual tubular cells [26]. From this point, multiple processes converge to drive cyst expansion—disrupted signalling, altered fluid transport, abnormal epithelial proliferation, and ECM remodelling [27]. Primary cilia are central to this story. When their mechanosensory function is lost, Ca^2+^ influx is impaired and intracellular cAMP rises compensatorily. Elevated cAMP then aberrantly activates MAPK/ERK signalling [28,29,30,31,32,33], driving a self-reinforcing cycle of epithelial proliferation, fluid secretion, and cyst enlargement, with dysregulated apoptosis as an additional contributor [26,27]. Arginine vasopressin (AVP), acting through the V2 receptor, is a key upstream driver of cAMP accumulation in collecting duct cells—the basis for tolvaptan’s mechanism: vasopressin type-2 receptor (V2R) antagonism reduces intracellular cAMP and slows renal enlargement and functional decline in at-risk patients [6].

***Fibrosis and ECM Remodelling*:** Like most progressive nephropathies, ADPKD is accompanied by interstitial fibrosis driven largely by TGF-beta overexpression and collagen accumulation [30,32]. Activin A, a TGF-beta superfamily member, is elevated in PKD cells and animal models, and its blockade attenuates cyst formation, pointing to an active role in disease progression [34,35,36]. ECM accumulation, changes in ECM-modifying enzyme composition, and heightened integrin-ECM crosstalk all promote aberrant proliferation and fibrogenesis [31,35], while alpha-smooth muscle actin (alpha-SMA)-positive myofibroblasts confirm ongoing fibrotic activity in ADPKD kidneys [35].

***Key Signalling Pathways***: mTOR is hyperactivated in cyst-lining cells in both human ADPKD and mouse models—reflected by elevated phospho-mTOR (Ser2448) and phospho-S6K (Thr389) [36]. Rapamycin works well in rodents, but the clinical story is different: two major randomized trials of sirolimus and everolimus failed to show meaningful benefit on kidney volume or function, most likely because drug concentrations within cystic epithelia were insufficient and compensatory pathway activation emerged [37,38]. This is an important lesson for any therapeutic strategy in ADPKD. AMPK, the cellular energy sensor, works in opposition to mTOR. It is consistently underactive in PKD epithelial cells, and restoring its activity—via metformin, for instance—simultaneously inhibits mTOR and reduces CFTR-dependent fluid secretion [39]. This dual effect makes AMPK a particularly attractive therapeutic target. Finally, cystic cells display a striking metabolic shift toward aerobic glycolysis—the so-called Warburg effect—abandoning oxidative phosphorylation to fuel rapid proliferation. This reprogramming is accompanied by mitochondrial dysfunction and elevated ROS, which amplify the inflammatory and fibrotic processes already described [40,41].

## 4. Polyphenols: Classification, Sources, and Biological Properties

Polyphenols are a structurally diverse class of plant-derived compounds defined by the presence of one or more hydroxyl groups (–OH) attached to aromatic rings. Based on their chemical structure, they are broadly classified into phenolic acids, phenolic alcohols, lignans, and flavonoids; the latter encompass several subclasses including anthocyanins, flavanols, flavones, flavanones, and stilbenes—of which resveratrol is the most extensively investigated [42]. To date, more than 8000 distinct polyphenolic compounds have been identified across the plant kingdom [43,44]. Dietary sources include fruits, berries, tea, red wine, leaves, stems, and seeds, with lower concentrations present in vegetables, legumes, and cereals [42,44]. The best-characterized biological property of polyphenols is their antioxidant activity, achieved through direct scavenging of free radicals and suppression of reactive oxygen species (ROS) generation, thereby protecting proteins, lipids, and nucleic acids from oxidative damage [43,45]. Beyond antioxidant effects, polyphenols modulate a broad spectrum of cellular pathways. Epidemiological and experimental evidence supports their role in reducing cardiovascular risk through inhibition of angiotensin-converting enzyme (ACE) activity, improvement of endothelial function, and attenuation of vascular inflammation [42,46]. They have also been shown to limit neurodegeneration and improve cognitive performance [47], reduce lipogenesis in metabolic-associated fatty liver disease [48], and exert beneficial effects in hyperglycaemic and dyslipidaemic disorders [49].

Of particular relevance to renal medicine, polyphenols exert renoprotective effects across a range of conditions, including diabetic nephropathy, hypertension-related kidney injury, and cardiorenal syndromes [1,2,50]. Cocoa flavanols have demonstrated vasculoprotective effects in patients on haemodialysis [51], and certain polyphenolic compounds have shown antitumoral properties in renal cell carcinoma [50].

Mechanistically, polyphenols converge on several interconnected stress-response pathways. They restore mitochondrial quality through SIRT1/PINK1/Parkin-mediated mitophagy, suppress NLRP3 inflammasome activation and NF-κB signalling, and attenuate cellular senescence markers including p53 and IL-6, as demonstrated in preclinical experimental models [2,51]. Emerging evidence also highlights their capacity to modulate the gut–kidney axis by favourably altering intestinal microbiota composition and reducing the production of pro-inflammatory uremic toxins, thereby indirectly limiting systemic oxidative stress [2].

A critical translational challenge for all polyphenolic compounds is their limited oral bioavailability, attributable to poor aqueous solubility, rapid hepatic metabolism, and extensive conjugation in the intestinal wall [43,44]. This pharmacokinetic limitation must be considered when interpreting preclinical data and designing clinical interventions, including in the context of ADPKD, where sustained tissue-level concentrations are likely required to modulate chronic cystogenic signalling cascades such as the mTOR, AMPK, and cAMP pathways. Following ingestion, these compounds undergo rapid conjugation in the intestinal wall, extensive first-pass hepatic metabolism, and—in many cases—poor aqueous dissolution, resulting in low and highly variable systemic concentrations. In the context of a chronic disease such as ADPKD, where sustained engagement of cystogenic signalling cascades is likely required for meaningful therapeutic effect, this pharmacokinetic constraint must be considered when interpreting preclinical findings and designing clinical interventions.

### 4.1. Compounds

Several natural and polyphenolic compounds have been investigated for their potential to delay ADPKD progression. The first systematic review of their mechanisms was published by Mahendran et al., 2021 [52] and subsequently updated by Zhang et al., 2023 [53]. The present review builds on this evidence base, integrating literature published after 2021 and extending the analysis to compounds not previously discussed in the ADPKD context.

#### 4.1.1. Resveratrol

Resveratrol (RSV, 3,4′,5-trihydroxystilbene) is found in grapes, berries, peanuts, Itadori tea (Polygonum cuspidatum), and red wine [46,53,54]. Its renoprotective properties are well established—antioxidant, anti-inflammatory, and endothelial-protective—and partly depend on nitric oxide signalling in models of nephrectomy and ischaemia–reperfusion [55,56,57,58].

In mesangial cells, RSV blocks PDGF-induced proliferation by preventing tyrosine phosphorylation at Y751 and Y716 on the PDGF receptor—the docking sites for PI3K and Grb2—and suppressing downstream Akt, ERK1/2, and c-Src [59]. Under hyperglycaemic conditions, it blocks mesangial fibronectin overexpression through JNK/NF-kB/NADPH oxidase/ROS pathway inhibition [60]. RSV also activates SIRT1 in the remnant kidney with consequent mitochondrial benefit [61], reduces diabetic renal injury through HRD1-mediated IGF-1R ubiquitination [62], and promotes SIRT1-dependent autophagy in diabetic nephropathy [63]. For ADPKD specifically, Wu et al. used the non-orthologous Cy/+ Han:SPRD rat to show that RSV reduced BUN, creatinine, the two-kidney/body weight ratio, macrophage infiltration, and the proliferation index. Mechanistically, it decreased cystic expression of MCP-1, TNF-alpha, and complement factor B and attenuated both NF-kB and mTOR activation via reduced p65 phosphorylation. The same effects were reproduced in human ADPKD cell lines and, in a zebrafish PKD model, RSV again suppressed both cyst growth and NF-kB activity [64]. What emerges is a compound that simultaneously tackles inflammation, proliferation, and mTOR—three core drivers of cystogenesis. The caveat is that the Cy/+ rat is not an orthologous ADPKD model, and no clinical data exist for RSV in ADPKD.

#### 4.1.2. Curcumin

Curcumin (diferuloylmethane) comes from the rhizome of Curcuma longa and carries an impressive preclinical record: antioxidant, anti-inflammatory, anticancer, and antifibrotic properties are all well documented [65,66]. In renal tubular cells, it alleviates TGF-beta1-induced apoptosis and modulates the circ_0008925/miR-204-5p/IL6ST axis to limit fibrosis [67]. In CKD models it reduces MCP-1, NF-kB, TNF-alpha, IL-1beta, COX-2, and caveolin-1, while upregulating the cytoprotective HO-1 and NEDD4, through TGF-beta/Smad, MAPK/ERK, and PPAR-gamma [68].

The problem is bioavailability. Standard oral curcumin produces negligible plasma and tissue concentrations [69], which has driven development of enhanced formulations. Theracurcumin suppresses NLRP3 inflammasome activation and attenuates CKD-associated cardiac fibrosis in rats [70]; the synthetic analogue C66 offers improved stability and blocks JNK2-driven pro-inflammatory and pro-oxidative responses in diabetic kidney and cardiovascular models [71].

In ADPKD, curcumin has been tested across multiple experimental systems. Leonhard et al. showed dose-dependent reduction of phospho-STAT3 and rpS6 in PKD1-deleted mice, with simultaneous interference across several cystogenic pathways [72]. In MDCK and embryonic kidney cyst models, it slowed cyst enlargement by reducing Ras, B-Raf, phospho-MEK, phospho-ERK, c-fos, and Egr-1 in forskolin-treated cells [73]. Since JAK2 is overexpressed in cyst-lining and interstitial cells in ADPKD, curcumin’s inhibition of JAK2 activity with consequent STAT3 dephosphorylation adds another relevant mechanism [74]. Combining curcumin with ginkgolide B—a terpenoid lactone from Ginkgo biloba—yielded synergistic inhibition in MDCK models, acting through EGFR/ERK1/2 and PI3K/mTOR [75,76].

Yet the only clinical trial—a randomized, double-blind, placebo-controlled study in 68 children and young adults with ADPKD—found no benefit on arterial dysfunction or kidney growth [77]. The most plausible explanation is bioavailability failure, not lack of biological activity. Short treatment duration and the inherent genetic heterogeneity of the study population likely compounded the problem. Better formulations and longer trials are the logical next step.

#### 4.1.3. Naringenin

Naringenin is a flavanone found predominantly in the peel and flesh of *Citrus* species, particularly grapefruit, oranges, and tangerines [78]. It exhibits antioxidant, antifibrotic, anti-inflammatory, antiviral, anticancer, and immunomodulatory properties across a broad range of experimental systems [79,80].

In renal tissue, naringenin attenuates doxorubicin-induced nephrotoxicity by downregulating NF-κB, TNF-α, and prostaglandin E2 [81]. Under hyperglycaemic conditions, it suppresses inflammation-mediated nitric oxide overproduction and enhances endogenous antioxidant capacity [82]. In animal models of diabetic nephropathy, naringenin reduces lipid peroxidation, increases superoxide dismutase and catalase activities, and attenuates TGF-β and IL-1β expression, while ameliorating microalbuminuria, hyperfiltration, and hyperglycaemia by reducing circulating and renal IL-1β, IL-6, TNF-α, and TGF-β [83,84]. Of note, naringenin enhances ATP synthase activity and SIRT1 expression—mechanistic effects of relevance to the metabolic and sirtuin-related pathway dysregulation characteristic of ADPKD.

Of direct relevance to cystogenesis, Waheed et al. demonstrated in Dictyostelium models and MDCK-derived cysts that naringenin inhibited tubular cell growth and cyst formation in a TRPP2 (polycystin-2)-dependent manner: crucially, knockdown of TRPP2 in MDCK cells conferred resistance to naringenin’s anti-cystogenic effect, directly implicating PC2 as the molecular mediator of this activity [85]. This establishes a mechanistically specific interaction with a core ADPKD protein, distinguishing naringenin from many polyphenols whose ADPKD-relevant actions operate exclusively on downstream inflammatory or metabolic pathways.

#### 4.1.4. Quercetin

Quercetin (3,3′,4′,5,7-pentahydroxyflavone) is a flavonol widely distributed in vegetables, fruits, tea, and wine [86,87]. Its antioxidant, anti-inflammatory, and antihypertensive properties are well characterized, with clinical evidence supporting kidney-protective effects in hypertensive patients [86,87,88,89,90].

In the kidney, quercetin modulates macrophage polarization by downregulating NF-κB p65 and IRF5 while inhibiting pro-inflammatory macrophage activation, thereby reducing extracellular matrix accumulation and interstitial fibrosis through TGF-β1/Smad2/3 pathway antagonism [91]. In diabetic kidney disease, it inhibits ferroptosis via Nrf2 activation [92], ameliorates renal lipid accumulation through the PPARA/PPARG-UCP1 axis [93], and prevents tubular epithelial apoptosis through PI3K/AKT pathway modulation [94].

In preclinical ADPKD models, quercetin inhibited cyst formation and enlargement in MDCK cells and murine embryonic kidney cultures and reduced the cystic index in PKD mice by suppressing PI3K/Akt and ERK phosphorylation; treatment also restored E-cadherin expression, which is significantly diminished in PKD kidneys [95]. These findings are mechanistically coherent with ADPKD pathogenesis, in which aberrant PI3K/Akt/ERK signalling drives cystic epithelial proliferation and E-cadherin loss disrupts cell–cell junctions. More recently, cellular senescence has been recognized as an active driver of ADPKD progression: senescence is increased in Pkd1 mutant mice and human ADPKD kidneys, and senescent cells promote cystogenesis through the Senescence-Associated Secretory Phenotype (SASP)-mediated induction of neighbouring cell senescence, epithelial proliferation, and fibroblast activation [96]. Treatment with the senolytic combination of dasatinib and quercetin delayed disease progression in Pkd1 mutant mice [96], identifying quercetin’s senolytic activity as a mechanistically distinct and clinically translatable action complementary to its anti-inflammatory and anti-proliferative effects.

#### 4.1.5. Cardamonin

Cardamonin is a chalcone polyphenol from the Zingiberaceae family—primarily Alpinia and Elettaria species—and stands out as one of the few compounds with direct experimental evidence in ADPKD [53].

He et al. showed that cardamonin simultaneously inhibits cyst growth and interstitial fibrosis in ADPKD preclinical models [97], acting through Wnt/beta-catenin and NF-kB suppression. This dual action matters: most current and experimental therapies address either cyst growth or fibrosis, but rarely both. Interstitial fibrosis is an independent driver of renal function decline in ADPKD and is inadequately targeted by tolvaptan or by compounds focused exclusively on the cAMP/mTOR axis. Cardamonin’s safety profile in other settings is encouraging, and validation in orthologous ADPKD models is the obvious next step.

#### 4.1.6. Gambogic Acid

Gambogic acid (GA) is a polyprenylated xanthone from the resin of Garcinia hanburyi. Taxonomically it sits at the edge of the polyphenol class, but its aromatic hydroxyl scaffold places it within the broader phenolic family. Anti-inflammatory, antioxidant, and antiproliferative properties have been documented across multiple systems [53].

Khunpatee et al. found that GA dose-dependently inhibited MDCK cyst growth and formation without cytotoxicity and suppressed proliferation in both MDCK and Pkd1 mutant cells [98]. Its mechanism is three-pronged: ERK1/2 inhibition, mTOR/S6K suppression, and AMPK activation. This is essentially the pharmacological logic of metformin applied through a different scaffold, with the added benefit of concurrent MAPK/ERK suppression. A single compound engaging all three nodes simultaneously is worth pursuing in orthologous models.

#### 4.1.7. EGCG and Green Tea Catechins

EGCG—epigallocatechin-3-gallate—is the main bioactive catechin in green tea and has well-documented renoprotective, antioxidant, anti-inflammatory, and antifibrotic properties, including in cardiorenal disease [2]. Direct ADPKD-specific evidence is currently lacking, but its mechanistic profile is highly relevant.

EGCG modulates the AMPK/mTOR axis [2], directly relevant to the metabolic reprogramming of ADPKD: AMPK activation suppresses both mTOR-driven anabolic signalling and Cystic Fibrosis Transmembrane conductance Regulator (CFTR)-mediated fluid secretion into cysts [39], while countering the aerobic glycolysis shift of cystic epithelia [40,41]. In renal models, EGCG suppresses NLRP3 inflammasome activation via the TXNIP/NLRP3/IL-1beta pathway, activates Nrf2/HO-1, and prevents tubular EMT through Nrf2-dependent mechanisms [2]. Its capacity to engage the SIRT1/PINK1/Parkin mitophagy axis provides a further link to the sirtuin biology elaborated by Carollo et al. in the cardiorenal context [1,2], which underpins part of the rationale for this review.

Given EGCG’s safety profile and the bioavailability improvements achievable with standardized green tea extract formulations, the case for testing it in orthologous ADPKD models is reasonable and should be pursued. Summary of polyphenolic compounds investigated are shown in Table 1.

## 5. Discussion

ADPKD’s therapeutic landscape is frustratingly narrow. Tolvaptan remains the only approved disease-modifying drug after decades of clinical research, and its use is complicated by hepatotoxicity risk, persistent aquaretic side effects, and cost—all of which limit real-world uptake [6]. The failure of mTOR inhibitors, despite powerful preclinical rationale, illustrated a key lesson: ADPKD is too complex for single-target therapy. The simultaneous dysregulation of NF-kB, mTOR, MAPK/ERK, PI3K/Akt, JAK/STAT, TGF-beta, and AMPK is not a series of independent parallel events—these pathways are interconnected, and blocking one node reliably activates compensatory ones [26,27].

This is precisely where polyphenols become interesting. Their defining pharmacological feature is not potency at a single target—it is breadth. The compounds reviewed here engage multiple cystogenic nodes simultaneously, at concentrations achievable through dietary or supplemental intake. Whether this translates into clinical benefit remains unknown, but the mechanistic logic is sound, and it represents a genuinely different therapeutic philosophy from the inhibitor-based approaches that have so far failed.

The most extensive ADPKD-specific preclinical data belong to resveratrol. Wu et al.’s work in Cy/+ rats and human ADPKD cell lines documented concurrent attenuation of NF-kB inflammation, mTOR activation, macrophage infiltration, and complement-mediated injury [64]. Notably, RSV reduced phospho-S6K in cystic kidneys, but this did not tightly correlate with reduced proliferation in vitro—suggesting that its anti-proliferative action runs primarily through NF-kB suppression and SIRT1-mediated metabolic regulation rather than direct mTOR inhibition [64], consistent with RSV’s established capacity to activate SIRT1 and NAD+-dependent pathways [1,2].

The metabolic angle deserves emphasis. Cystic epithelia shift from oxidative phosphorylation to aerobic glycolysis to fuel their high proliferative demands [40,41], and AMPK activity is consistently reduced in this context [39]. Restoring AMPK function simultaneously suppresses mTOR, reduces fluid secretion, and counters the glycolytic shift—making it one of the most coherent single-target strategies available for ADPKD. Several polyphenols converge on this node: resveratrol through SIRT1/NAD+ activation, EGCG through direct AMPK engagement, and gambogic acid through AMPK restoration alongside ERK and mTOR suppression [64,98]. None of these replicate tolvaptan’s mechanism, raising the possibility of genuine complementarity and potentially additive effects in combination.

Naringenin and quercetin bring distinct mechanisms that expand the scope of what polyphenols can offer in ADPKD. Naringenin’s anti-cystogenic action depends on functional TRPP2/polycystin-2—cells lacking PC2 are resistant to its effect [85]. This is not a downstream anti-inflammatory maneuver; it is a direct interaction with a core disease protein. Quercetin, meanwhile, now has evidence supporting a senolytic role: senescence accumulates in Pkd1 mutant kidneys and actively promotes cystogenesis through SASP, and the dasatinib-quercetin combination delayed disease progression in this model [96]. These findings suggest that polyphenols can engage disease-driving mechanisms that current pharmacological approaches do not address at all.

The limitations of the current evidence base must, however, be stated clearly. Most data come from non-orthologous or cAMP-driven models—the Cy/+ rat and MDCK system are experimentally convenient but do not carry PKD1 or PKD2 mutations. Even Pkd1 conditional knockout models, while more relevant, do not fully reproduce the slow, heterogeneous progression of human disease. The Pkd1RC/RC mouse—a hypomorphic model that more faithfully mirrors human disease trajectory—has been used in few of the studies reviewed here, and validation in this system should be a prerequisite before any compound advances toward clinical consideration.

The curcumin RCT is a cautionary tale that illuminates the bioavailability problem. Despite compelling preclinical data across multiple ADPKD models, standard curcumin produced no clinical benefit in a well-designed trial [77]. The most parsimonious explanation is that conventional oral curcumin simply does not reach the kidney at concentrations required to engage its targets. Enhanced formulations—theracurcumin, nanoparticle systems, and phospholipid complexes—consistently outperform standard preparations in preclinical and early clinical settings [70,71]. Any future polyphenol trial in ADPKD should treat bioavailability as a primary design constraint, not an afterthought.

The combination question is also worth raising. Curcumin and ginkgolide B produced synergistic effects in MDCK models through complementary pathway targeting [76], and the mechanistic profiles of the compounds reviewed here are largely non-overlapping: RSV and cardamonin target NF-kB, naringenin targets polycystin-2 directly, gambogic acid and EGCG restore AMPK activity, quercetin clears senescent cells, and curcumin suppresses JAK/STAT and mTOR. A rationally designed combination could, in principle, provide the kind of multi-pathway coverage that the disease seems to require—though whether such combinations can be safely formulated and clinically delivered remains an open question that warrants systematic investigation. Finally, it should be acknowledged that parameters of direct clinical relevance—including compound-specific adverse effect profiles, potential drug interactions, and therapeutically effective dose ranges in the context of ADPKD—remain to be systematically characterized for most of the compounds reviewed here. With the exception of curcumin, none have been evaluated in disease-specific clinical trials, and extrapolation from studies in other conditions carries inherent limitations. The bioavailability data reviewed here, and the formulation strategies under development, nonetheless represent a necessary first step toward defining pharmacologically realistic dosing frameworks for future interventional studies.

## 6. Conclusions and Future Perspectives

The evidence reviewed here supports polyphenols as mechanistically coherent adjunctive candidates for ADPKD, capable of simultaneously targeting multiple cystogenic cascades—NF-κB inflammation, mTOR/S6K signalling, MAPK/ERK proliferation, TGF-β fibrogenesis, and AMPK-dependent metabolic reprogramming—a multi-target profile that distinguishes them from the single-pathway inhibitors that have failed clinical translation in ADPKD to date. Among the compounds reviewed, resveratrol, curcumin, naringenin, and quercetin have demonstrated direct anti-cystogenic activity in ADPKD-relevant models, while cardamonin and gambogic acid represent emerging candidates with promising preclinical data. The connection between polyphenols, sirtuin activation, and NAD^+^-dependent metabolic regulation further strengthens the rationale for their investigation in ADPKD, where mitochondrial dysfunction and Warburg-like metabolic reprogramming are increasingly recognized as core disease features [1,2,40,41].

The current evidence base is nonetheless substantially limited by its reliance on non-orthologous preclinical models and the absence of pharmacokinetically validated formulations.

The only randomized controlled trial of a polyphenol in ADPKD—evaluating curcumin in children and young adults—yielded a negative result, most likely attributable to inadequate bioavailability rather than lack of intrinsic activity [77]. Future research should prioritize validation in orthologous Pkd1/Pkd2 knockout models, development of nanoformulations or structural analogues with improved pharmacokinetic profiles, and investigation of rational combinations—including polyphenol-tolvaptan co-administration and the senolytic dasatinib-quercetin strategy—that exploit the mechanistic complementarity among compounds targeting distinct cystogenic nodes [76,96]. The identification of reliable biomarkers of target engagement will be equally essential to support the design of future clinical trials in this population.

## Figures and Tables

**Table 1:** Summary of polyphenolic compounds investigated in ADPKD: chemical class, molecular targets, key findings, and level of evidence.

Compound	Chemical Class	ADPKD-Relevant Pathway Targets	Key Preclinical Findings	Experimental Model	Clinical Evidence
Resveratrol [64]	Stilbene	NF-kB ↓, mTOR ↓, SIRT1 ↑, ERK ↓, MCP-1 ↓, CFB ↓	Reduced BUN, creatinine, cystic index; ↓ macrophage infiltration and proliferation index; ↓ NF-kB p65 phosphorylation	Cy/+ Han:SPRD rats; human ADPKD cell line; zebrafish PKD model	None in ADPKD
Curcumin [70,71,72,74,75]	Diarylheptanoid	STAT3 ↓, mTOR/S6K ↓, JAK2 ↓, ERK ↓, PI3K ↓, EGFR ↓	↓ phospho-STAT3 and rpS6; inhibition of cyst enlargement via Ras/B-Raf/ERK; ↓ JAK2/STAT3; synergistic effect with ginkgolide B	PKD1-KO mice; MDCK; murine embryonic kidney	No benefit on vascular dysfunction (RCT, n = 68, 2022) [77]
Naringenin [85]	Flavanone	TRPP2/PC2 (direct modulation), Nrf2 ↑, SIRT1 ↑, TGF-b ↓	TRPP2-dependent inhibition of cyst growth; resistance upon PC2 knockdown	Dictyostelium; MDCK-derived cysts	None in ADPKD
Quercetin [95,96]	Flavonol	PI3K/Akt ↓, ERK ↓, E-cadherin ↑, p16Ink4a senolytic activity	↓ cystic index; restored E-cadherin expression; delayed PKD progression via D + Q senolytic treatment	MDCK; Pkd1 mutant mice	None in ADPKD
Cardamonin [97]	Chalcone	Wnt/b-catenin ↓, NF-kB ↓, TGF-b ↓	Inhibition of cyst growth and interstitial fibrosis; dual anti-cystogenic and antifibrotic activity	ADPKD preclinical models	None
Gambogic Acid [98]	Xanthonoide	ERK1/2 ↓, mTOR/S6K ↓, AMPK ↑	Dose-dependent inhibition of MDCK cyst growth and formation; ↓ cell proliferation in Pkd1 mutant cells	MDCK; Pkd1 mutant cells	None
EGCG [1,2]	Flavanol (catechin)	AMPK ↑, mTOR ↓, Nrf2/HO-1 ↑, NF-kB ↓, NLRP3 ↓, SIRT1/PINK1/Parkin ↑	Renoprotective and anti-inflammatory effects in multiple kidney disease models; mechanistic convergence on ADPKD-relevant pathways	Multiple renal models (DKD, hypertension, CRS)	Limited; no ADPKD-specific trials

BUN: blood urea nitrogen; CFB: complement factor B; CRS: cardiorenal syndrome; D + Q: dasatinib plus quercetin; DKD: diabetic kidney disease; EGCG: epigallocatechin-3-gallate; MDCK: Madin–Darby canine kidney; RCT: randomized controlled trial.

## Data Availability

No new data were created.

## Abbreviations

ACE	Angiotensin-Converting Enzyme
ADPKD	Autosomal Dominant Polycystic Kidney Disease
AMPK	AMP-Activated Protein Kinase
AVP	Arginine Vasopressin
BUN	Blood Urea Nitrogen
cAMP	Cyclic Adenosine Monophosphate
CFB	Complement Factor B
CFTR	Cystic Fibrosis Transmembrane Conductance Regulator
CKD	Chronic Kidney Disease
CRS	Cardiorenal Syndrome
D + Q	Dasatinib plus Quercetin
DKD	Diabetic Kidney Disease
ECM	Extracellular Matrix
EGCG	Epigallocatechin-3-Gallate
EGFR	Epidermal Growth Factor Receptor
EMT	Epithelial–Mesenchymal Transition
ERK	Extracellular signal-Regulated Kinase
FDA	Food and Drug Administration
GA	Gambogic Acid
JAK2	Janus Kinase 2
MAPK	Mitogen-Activated Protein Kinase
MCP-1	Monocyte Chemoattractant Protein-1
MDCK	Madin-Darby Canine Kidney
mTOR	Mechanistic Target of Rapamycin
NAD+	Nicotinamide Adenine Dinucleotide
NF-κB	Nuclear Factor Kappa-Light-Chain-Enhancer of Activated B Cells
NLRP3	NOD-Like Receptor Thermal Protein Domain Associated Protein 3
PC1	Polycystin-1
PC2	Polycystin-2
PDGF	Platelet-Derived Growth Factor
PI3K	Phosphoinositide 3-Kinase
PKD1	Polycystic Kidney Disease gene 1
PKD2	Polycystic Kidney Disease gene 2
PPAR-γ	Peroxisome Proliferator-Activated Receptor gamma
RCT	Randomized Controlled Trial
ROS	Reactive Oxygen Species
RSV	Resveratrol
SASP	Senescence-Associated Secretory Phenotype
SIRT1	Sirtuin 1
SMA	Smooth Muscle Actin
STAT3	Signal Transducer and Activator of Transcription 3
TGF-β	Transforming Growth Factor Beta
TNF-α	Tumour Necrosis Factor Alpha
TRP	Transient Receptor Potential
TRPP2	Transient Receptor Potential Polycystin 2
V2R	Vasopressin type-2 Receptor
